# Pulmonary Mucormycosis: An Interesting Case of Rhizopus Mucormycosis

**DOI:** 10.7759/cureus.16210

**Published:** 2021-07-06

**Authors:** Chijioke D Ukoha, Nicholas Nguyen

**Affiliations:** 1 Internal Medicine, Methodist Dallas Medical Center, Dallas, USA

**Keywords:** pulmonary, mucormycosis, rhizopus, mucor, zygomycosis

## Abstract

We report a case of a 57-year-old Vietnamese gentleman who presented with chest pain and shortness of breath for four weeks. The patient had a history of diabetes mellitus and kidney transplant in the past year and was currently on immunosuppressive agents. The patient's condition worsened despite broad-spectrum antibiotics, so amphotericin was added. Further evaluation with bronchoscopy and transbronchial biopsy was suggestive of *Rhizopus* mucormycosis. Despite antifungal therapy, his condition worsened, resulting in multi-organ failure and eventual mortality.

## Introduction

Mucormycosis is defined as a serious, sometimes fatal, fungal infection that originates from the fungi order *Mucorales*. In humans, the genera most commonly causing mucormycosis are *Mucor*, *Rhizopus*, and *Rhizomucor*. These organisms are commonly found in decaying vegetables or in soil. Mucormycosis tends to infect individuals with hematopoietic malignancies, uncontrolled diabetes mellitus, or those that are immunocompromised [1]. Mucormycosis has a wide spectrum of presentations that include rhinocerebral, pulmonary, cutaneous, gastrointestinal, disseminated, and uncommon presentations [2]. Pulmonary mucormycosis (PM) is the second most common mucormycosis syndrome after nasal. PM is a rapidly progressing disease that can be fatal if left untreated [3]. Patients contract PM through inhalation of spores, which infect bronchioles and alveoli or by hematogenous or lymphatic spread [4]. The use of antifungal agents (e.g., amphotericin B [AMB]), in combination with surgical debridement, is the ideal treatment for PM patients; early treatment drastically affects survival [5]. Here, we report a case of PM caused by an invasion of the fungi genus *Rhizopus.*

## Case presentation

The patient was a 57-year-old Vietnamese male with a significant past medical history of end-stage renal disease (status: post-deceased donor kidney transplant January 2020), coronary artery disease (status: post-coronary artery bypass graft in 2012), diabetes mellitus type 2, hypertension, and a remote history of tuberculosis in 2000. He initially presented with a four-week history of persistent left-side chest pain. A prior myocardial perfusion scan was negative except for a fixed defect that was new from prior studies. The patient noted significant chest wall tenderness to the slightest touch. He reported minimal relief with tramadol, acetaminophen, gabapentin, and muscle relaxers. The patient also had associated dyspnea and red-tinged urine with an onset three days prior to arrival. His family also endorsed a new-onset cough with red-tinged sputum. A repeat outpatient tuberculosis test was negative. Of note, the patient had prior methicillin-susceptible *Staphylococcus aureus* scalp lesions and prior cytomegalovirus viremia in July 2020 that was treated with Valcyte. Also, the family reported that the patient had been in his garden more than usual prior to his admission. Important home medications included amiodarone, apixaban, prednisone, fludrocortisone, and mycophenolate sodium. 

The physical examination revealed that the patient was afebrile, but tachycardic with a pulse of 105 bpm, hypertensive with a blood pressure of 145/75 mmHg, and breathing well with oxygen saturation of 98% on room air. He was an ill-appearing male. The patient’s neck was tender to palpation and swollen anteriorly. His oral mucosa was positive for thrush. A cardiopulmonary examination noted diminished breath sounds bilaterally with left-sided crackles and pleural rub. A musculoskeletal examination revealed exquisite tenderness to palpation along the left T4 to T10 dermatome. Laboratory studies showed a white blood cell count of 3.9 x 10^9^/L, hemoglobin level of 12.12 g/dL, troponin level of 0.22 ng/mL, creatinine 2.24 mg/dL, calcium level of 10.5 mg/dL, aspartate transaminase level of 48 U/L, alanine aminotransaminase level of 33 U/L, lactate dehydrogenase level of 1418 IU/L, ferritin level of 5000 ng/mL, thyroid-stimulating hormone level 0.06 uIU/mL, free T4 6.43 ng/dL, and procalcitonin level of 2.0 ng/mL. A chest computed tomography (CT) scan revealed pneumonic infiltrate with trace pleural fluids in the left lower lobe and posterior portions of the left upper lobe, small mediastinal lymph nodes, and an enlarged right lobe of the thyroid with thyroid nodularity on the right lobe. A thoracic and lumbar CT was negative for osseous abnormalities. A soft tissue neck CT showed a dominant 2.8 × 2.3 × 3.4 cm nodule within the right lobe of the thyroid gland. 

The patient was admitted for further evaluation of his anterior neck swelling, T4 through T10 dermatomal pain concerning for spinal stenosis, and an acute kidney injury. He was started on broad-spectrum antibiotics including vancomycin, cefepime, metronidazole, and fluconazole. Mycophenolate sodium was discontinued due to concerns of infection, but prednisone was increased as per recommendations from Endocrinology who stated this would help. Amiodarone was also discontinued given concerns of this being the culprit of the patient's thyrotoxicosis, and the patient was started on methimazole. 

Overnight, the patient developed acute hypoxic respiratory failure (requiring increased oxygen), hypotension, and worsening chest x-ray findings concerning for pneumonia. He was subsequently transferred to the ICU for closer monitoring. Given the patient was clinically deteriorating, he was emergently intubated, antibiotics were transitioned to meropenem given his acute neutrophilic count was 750 neutrophils/mcL, and antifungal coverage was transitioned to Voriconazole. Infectious labs, such as urine *Legionella*, urine *Streptococcus pneumoniae*, COVID-19 test, and respiratory viral panel, were negative. 

**Figure 1:**
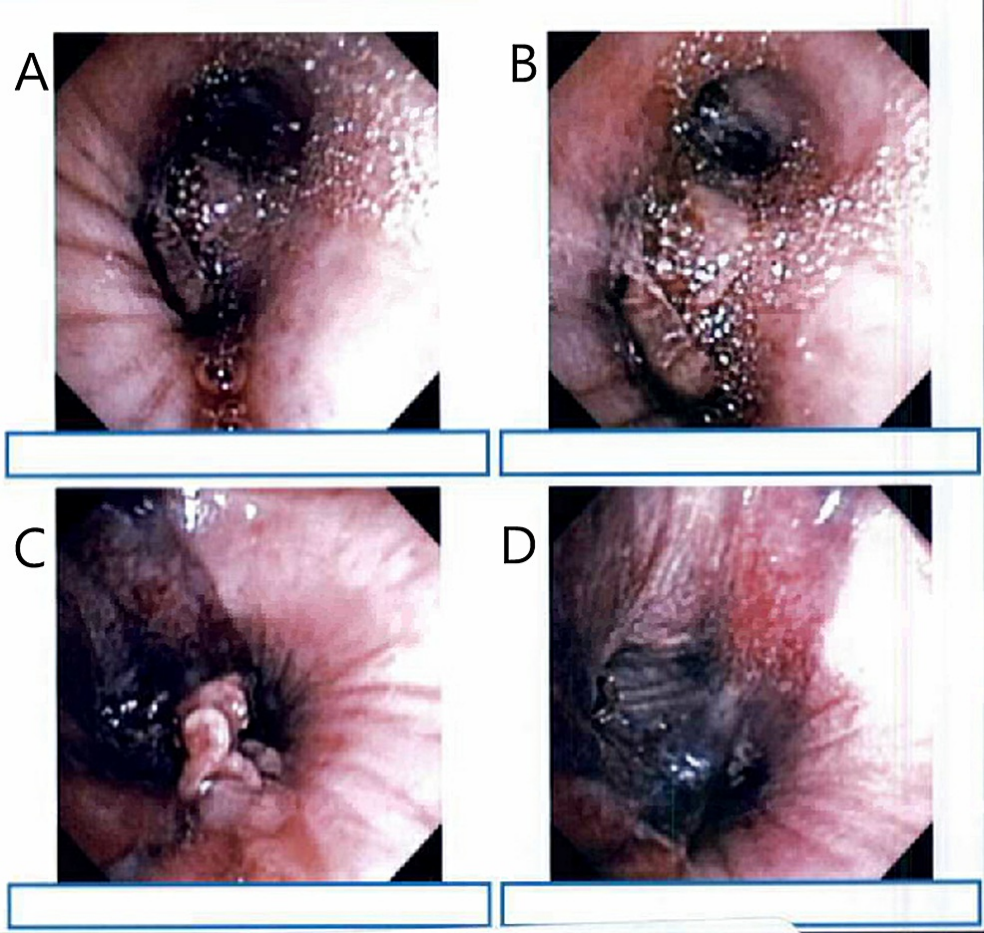
Bronchoscopy depicting necrotizing tissue concerning for pulmonary mucormycosis

The patient underwent a bronchoscopy that was concerning for a devascularized necrotizing lung infection with vascular affinity, along with alveolar hemorrhage within the left lung. Voriconazole was transitioned to AMB for broader antifungal coverage and the prednisone dose decreased given concern for fungal infection. A bronchoalveolar lavage remained negative for yeast or fungus. Continuous renal replacement therapy was initiated given oliguria. The patient underwent a repeat bronchoscopy that demonstrated moldy filamentous growth at the level of the left upper lobe bronchus (Figure 1). Fungal cultures from a bronchial biopsy grew *Rhizopus*. 

The patient shortly developed shock on day 3 secondary to sepsis and atrial fibrillation with rapid ventricular response, and was initiated on pressor therapy. The patient continued to clinically decline and required cardiopulmonary resuscitation, in which return of spontaneous circulation was obtained. The family was notified and they decided to institute a do-not-resuscitate order and withdrew life support.

## Discussion

Mucormycosis is characterized by tissue necrosis resulting from angioinvasion and subsequent thrombosis [2]. Increased iron uptake by the fungi promotes fungal growth and subsequent worsening infection [5]. Diabetic patients exhibit increased risk of infection due to impaired transferrin binding resulting in increased serum iron [5]. 

PM patients usually present with nonspecific symptoms, such as prolonged high-grade fever that persists despite broad-spectrum antibiotics, cough, hemoptysis, dyspnea, and chest pain [2]. Radiological findings vary; however, common findings are cavitations, infiltration, posterior tracheal band thickening, consolidation, nodules, atelectasis, hilar or mediastinal lymphadenopathy, effusion, and even normal findings [2]. PM can be difficult to diagnose as patient presentation is similar to patients with *Aspergillus* infections. Definitive distinction of PM can be made with sputum or bronchoalveolar lavage (BAL). *Mucorales *specimens demonstrate large (10-30 mm) ribbon-like twisted aseptated hyphae as opposed to *Aspergillus*, which demonstrate dichotomous branching, with septated hyphae 5-10 mm in diameter [6].

The mainstay PM treatment is intravenous AMB. However, a lipid formulation of AMB delivered at a higher dose through aerosolized therapy is preferred as it is associated with decreased risk of nephrotoxicity. The use of antifungal agents in combination with surgical debridement is ideal as it drastically improves survival [5]. The overall mortality rate in patients with PM is high (76%), so it is imperative to have early diagnosis and treatment [2]. 

A major component for the successful treatment of PM is managing detrimental risk factors (e.g., diabetes or immunosuppression) present in the patient [1]. In this case, the patient was initially started on Voriconazole with antifungal coverage before being switched to AMB after no improvement on broad-spectrum antibiotics. Unfortunately, due to the rapid course of PM, the patient outcome was unfavorable.

## Conclusions

Mucormycosis is a deadly, rapidly progressing fungal infection mostly seen in immunocompromised individuals, such as those with diabetes mellitus, hematologic malignancies, or patients on immune inhibitors. PM is the second most common form of mucormycosis. These patients present with nonspecific symptoms, so prompt diagnosis is imperative for survival. Definitive diagnosis is through sputum or BAL. The mainstay of treatment is AMB along with surgical debridement. Immediate treatment is necessary to ensure better prognosis. Our case highlights the need for thorough evaluation and rapid treatment when a patient presents with symptoms that indicate possible PM.
